# Locking down the Impact of New Zealand’s COVID-19 Alert Level Changes on Pets

**DOI:** 10.3390/ani11030758

**Published:** 2021-03-10

**Authors:** Fiona Esam, Rachel Forrest, Natalie Waran

**Affiliations:** 1 Companion Animals New Zealand, Wellington 6141, New Zealand; 2 Eastern Institute of Technology, Hawke’s Bay 4112, New Zealand; rforrest@eit.ac.nz (R.F.); nwaran@eit.ac.nz (N.W.)

**Keywords:** companion animal, COVID-19, exercise, lockdown, New Zealand, pandemic, pet, play, welfare, wellbeing

## Abstract

**Simple Summary:**

This investigation explored the impact of the COVID-19 pandemic lockdown and following alert levels on pets in New Zealand. Pet owners were surveyed during the last week of the first Alert Level 4 lockdown (highest level of restrictions) and then three months later during Alert Level 1 (lowest level of restrictions). During lockdown, just over half of those surveyed thought that their pet’s wellbeing was better than usual, and most owners could list at least one benefit of lockdown for their pets. These included more company, play and exercise. Owners expressed that they were concerned about their pet’s wellbeing after lockdown, with pets missing company/attention and separation anxiety being major themes. The Alert Level 1 survey indicated that owners continued to play with their pets more but that higher levels of exercise were not maintained. Just over one-third of owners took steps to prepare their pets to transition out of lockdown. The results indicate that pets may have enjoyed improved welfare during lockdown due to the possibility of increased human-pet interaction. The steps taken by owners to prepare animals for a return to normal life may enhance pet wellbeing long-term if maintained.

**Abstract:**

The influence of the COVID-19 pandemic on human-pet interactions within New Zealand, particularly during lockdown, was investigated via two national surveys. In Survey 1, pet owners (n = 686) responded during the final week of the five-week Alert Level 4 lockdown (highest level of restrictions—April 2020), and survey 2 involved 498 respondents during July 2020 whilst at Alert Level 1 (lowest level of restrictions). During the lockdown, 54.7% of owners felt that their pets’ wellbeing was better than usual, while only 7.4% felt that it was worse. Most respondents (84.0%) could list at least one benefit of lockdown for their pets, and they noted pets were engaged with more play (61.7%) and exercise (49.7%) than pre-lockdown. Many respondents (40.3%) expressed that they were concerned about their pet’s wellbeing after lockdown, with pets missing company/attention and separation anxiety being major themes. In Survey 2, 27.9% of respondents reported that they continued to engage in increased rates of play with their pets after lockdown, however, the higher levels of pet exercise were not maintained. Just over one-third (35.9%) of owners took steps to prepare their pets to transition out of lockdown. The results indicate that pets may have enjoyed improved welfare during lockdown due to the possibility of increased human-pet interaction. The steps taken by owners to prepare animals for a return to normal life may enhance pet wellbeing long-term if maintained.

## 1. Introduction

The World Health Organisation declared Corona Virus disease 2019 (COVID-19) caused by the Severe Acute Respiratory Syndrome coronavirus 2 (SARS-CoV-2) a pandemic on 11 March 2020,

*WHO has been assessing this outbreak around the clock and we are deeply concerned both by the alarming levels of spread and severity, and by the alarming levels of inaction. We have therefore made the assessment that COVID-19 can be characterized as a pandemic* [1].

In New Zealand, the first COVID-19 case was reported on the 28th of February 2020, and the country’s borders were closed to all non-residents from the 19th of March 2020 [2]. The Government introduced the 4-tiered Alert Level system to help combat COVID-19 on the 21st of March 2020, which in brief is Alert Level 4—Lockdown, Alert Level 3—Restrict, Alert Level 2—Reduce, and Alert Level 1 Prepare [3]. A nationwide lockdown (Alert Level 4) began at 11.59 pm on the 25th of March 2020, requiring anyone not deemed an essential worker to go into self-isolation with their household or ‘bubble’ until the 27th of April 2020 11:59 pm at which time the country moved to Alert Level 3, partially lifting some of the lockdown restrictions [2]. On the 13th of May 2020, New Zealand moved to Alert Level 2 lifting the rest of the lockdown restrictions while maintaining physical distancing and gathering size limits [2]. On the 8th of June 2020, the Ministry of Health reported that there were no active cases of COVID-19 in New Zealand, which moved to Alert Level 1 removing all remaining restrictions except border controls [2].

Many studies have explored the impact of quarantine and self-isolation due to disease outbreaks (including the COVID-19 pandemic) on humans for reviews see [4,5,6], but few studies have examined the impact on our companion animals (pets). Recent studies have reported that while pets provided their owners with a positive presence and support during the COVID-19 pandemic, some pets also showed stress-related behavioural changes due to the disruptions to their usual environment and routine [7,8,9,10]. Emerging pet behavioural issues along with other unique hardships pet owners face due to COVID-19 lockdowns (eg lack of access to animal supplies and services, exacerbated financial/economic issues) increases the risk of inadequate care and relinquishment or abandonment, resulting in negative impacts on the well-being of both the owner and pet [7,11]. This study investigated the impact of the nationwide lockdown on pet wellbeing in New Zealand as perceived by pet owners and discusses strategies to mitigate issues experienced by pet owners and to reinforce positive human behavioural changes that strengthen the human-animal bond, thus improving both animal and human wellbeing. Given that there is evidence that positive relationships with companion animals can be beneficial to the health and wellbeing of their owner and mitigate some of the detrimental effects of the COVID-19 lockdowns [7,8,9,10], this One Welfare approach promotes healthy communities during a global pandemic.

## 2. Materials and Methods

Data Collection: Online questionnaires were used to collect a convenience sample of respondents. The questionnaires used in Survey 1 and 2 were developed by Companion Animals New Zealand (CANZ) in consultation with One Welfare researcher Professor Natalie Waran from the Eastern Institute of Technology, Hawke’s Bay, New Zealand. Survey 1 was designed to inform CANZ’s social media information inventions to address pet owner concerns during and after the Alert Level 4 lockdown that were highlighted by the data collected. Both questionnaires included multiple-choice, Likert-like scale, and free text questions. A full copy of the questionnaires used for each survey can be found in the supplementary material (Figures S1 and S2). The questionnaires were administered online by distributing the survey link via social media and other networks. Survey 1 was open from the 24th of April to the 30th of April 2020, while Survey 2 was open from the 9th of July to the 28th of July 2020. The respondent inclusion criteria were New Zealand resident, pet owner and over the age of 18. Survey completion was anonymous and therefore it could not be determined if a person had completed both surveys or not.

Data Analysis: The quantitative data analysis was performed using SPSS Statistics Version 25 [12]. The data have been described using frequencies, percentages. Where appropriate, column proportions were compared using z tests (independent proportions) to identify any significant differences. All statistical analyses were performed with a significance level of 0.05.

The qualitative data were either analysed for emergent themes by at least two of the authors independently and then collaboratively categorised using a general inductive approach [13] or analysed using conventional content analysis [14]. Using content analysis, the presence (frequency) of reoccurring descriptions or concerns were quantified.

## 3. Results

### 3.1. Survey 1—During Alert Level 4 (Lockdown)

The survey was completed by 686 respondents. Table 1 summarises the percentage of pet type/s owned.

In 2020, cats are reported to be the most popular companion animal in New Zealand being part of 41% of households, with dogs being part of 34% [15], therefore, the convenience sample obtained does not appear representative of New Zealand pet owners.

Respondents were asked on a scale of 1 to 5 if they were spending more time at home during lockdown (1 = No more than normal, 5 = Far more than before). The results are shown in Table 2, with 82.5% (n = 566) of respondents selecting 4 or 5 indicating the adults in the household were spending a lot more time at home compared to their pre-lockdown normal. Thirty-two percent of the respondents (32.4%, n = 222) also had children at home during lockdown that would have normally been at school.

When asked if respondents felt that their pets had experienced any positive benefits from lockdown, 681 answered and 84.0% (n = 572) responded affirmatively. The themes that emerged from the perceived benefits for their pets described by the respondents were: differences between cats and dogs, for example, “the dog likes having me home but the cats couldn’t care less”; having company (subthemes: fewer movement restrictions such as not being crated or locked outside, more mental stimulation); more/better quality attention (subthemes: exercise, play, training, physical affection, grooming, treats, health surveillance/care, bonding), environment benefits (subthemes: fewer cars on road/less chance on being hit, less outside noise for example from vehicles and roadworks, more movement freedoms, access to heated areas on cold days).

When asked if respondents felt that their pets had experienced any negative impacts from lockdown, 681 answered, and 52.1% (n = 355) of respondents felt their pets had. The following themes emerged from the respondent explanations: Reduced quality of dog exercise (subthemes: less socialisation, restricted to on lead, restricted exercise areas/more usage by community members); disrupted routines (subthemes: less time to themselves, interrupted sleep/relaxation, excess attention, no car rides); environment changes (extra inside noise, arguing humans); health concerns (subthemes: restricted access to animal services and products, overfeeding, excess exercise injuries, more anxiety/stress, less money to provide for animal needs).

Using a Likert-like scale (1 = wellbeing is far worse, 5 = wellbeing is much better), respondents were asked to rate pets’ wellbeing. The results are shown in Table 3, with 54.7% (n = 375) of respondents selecting 4 or 5 indicating better wellbeing during lockdown while 7.4% (n = 51) of selected 1 or 2 indicating they though their pets’ wellbeing was better pre-lockdown. No significant differences were detected between cat and dog owners.

Of the 680 respondents that answered the question “Have you noticed any changes in your pets’ behaviour during lockdown?”, 56.9% (n = 387) responded that they had. Content analysis revealed that being more needy/clingy was the most often described behavioural change (n = 75). This was followed by pets being more affectionate (n=33), calmer/more relaxed (n = 30), and happier (n = 21). Conversely, some pets became more nervous/anxious/worried/stressed (n = 26), made more noise such as barking, whining, meowing (n = 30) and were more demanding (n = 17).

For most of the respondents (74.1%, n = 508) veterinary care was not required for any of their pets during the Alert Level 4 lockdown. Veterinary care for at least one of their pets was obtained by 26% (n =178) of the total respondents, with 30.3% (n = 54) of the consultations occurring via phone or email.

Respondents were asked on a scale of 1 to 5 how much exercise had their pets been getting during lockdown compared with pre-lockdown (1 = A lot less, 5 = A lot more). The results are shown in Table 4, with 44.8% (n = 307) of respondents selecting 4 or 5 indicating their pets got more exercise during lockdown while 12.5% (n = 86) selected 1 or 2 indicating their pets got more exercise pre-lockdown. Similar results are obtained when the data from only those respondents owning dogs is analysed. Most of the respondents (84.4%, n = 579) felt that the guidelines for dog walking during lockdown were reasonable, with 7.7% (n = 53) of respondents being unsure and 7.9% (n = 54) feeling the guidelines were not reasonable.

Using a Likert-like scale (1 = a lot less, 5 = a lot more), respondents were asked how much time they spent playing with their pets compared to pre-lockdown. The results are shown in Table 5, with 61.7% (n = 423) of respondents selecting 4 or 5 indicating they played more with their pets during lockdown while 2.3% (n = 16) of selected 1 or 2 indicating they played with their pets less.

**Figure 1:**
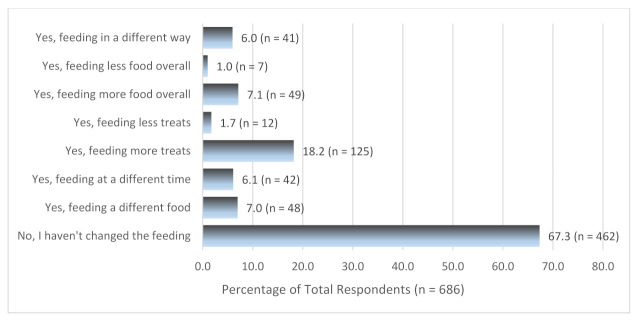
Feeding routine changes during lockdown.

Figure 1 shows how respondents changed how they feed their pets during the Alert Level 4 lockdown. Most of the respondents (67.3%, n = 462) did not change the way they feed their pets during lockdown. Eighteen percent of the respondents (18.2%, n = 125) reported giving their pets more treats during lockdown but only 7.1% (n = 49) thought they were feeding more food overall.

Of the 682 respondents that answered the question “Are you concerned about the future wellbeing of your pets after lockdown?”, 40.3% (n = 275) expressed that they were. Content analysis identified that the most commonly described concerns were that pets would miss having company and attention (n = 100) and experience separation anxiety (n = 83). Other common concerns included access to animal or veterinary services and products (n = 32) and issues with dog socialisation around other animals and people (n = 23). Sixty-four (n = 437) percent of the 682 respondents expressed that there were concerned about the future wellbeing of New Zealand pets generally after lockdown. The respondents’ primary concerns were separation anxiety (n = 125) along with loneliness and boredom (n = 104), the impact of financial hardship on caring for animals (n = 56), an increase in unwanted and inadequately cared for animals including pets that were acquired during lockdown and unwanted kittens and puppies due to a lack of desexing during lockdown (n = 47) and a decrease in exercise levels especially for dogs (n = 22).

### 3.2. CANZ Social Media Interventions

**Figure 2:**
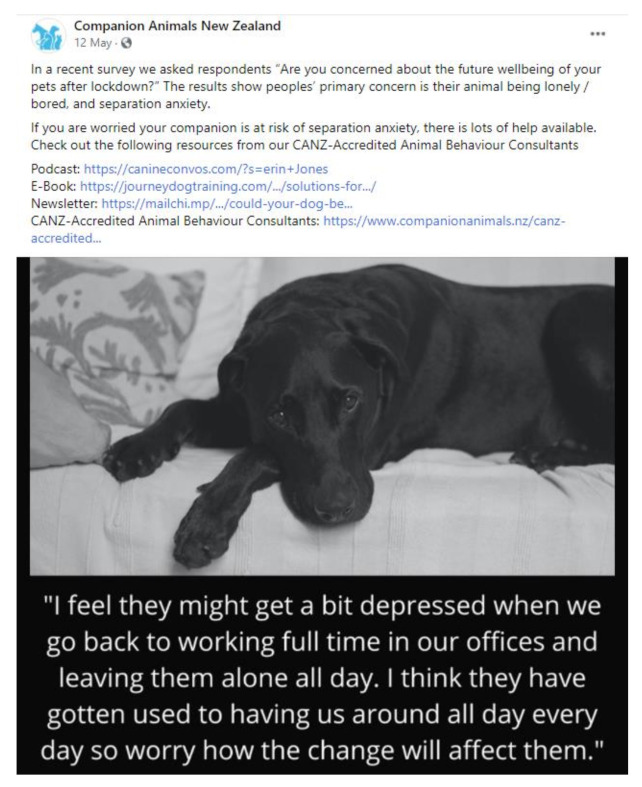
An example of a Facebook post by Companion Animals New Zealand addressing a common concern regarding post-lockdown among pet owners and providing links to appropriate resources.

**Figure 3:**
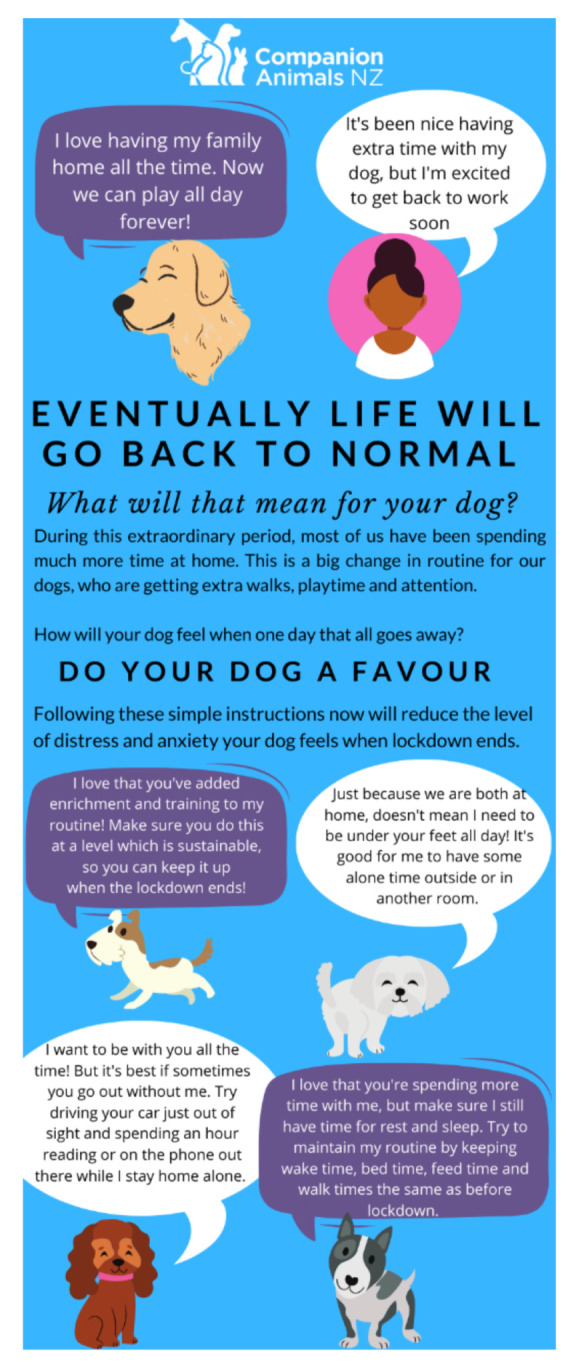
The most engaged with Facebook post by Companion Animals New Zealand providing information to reduce separation anxiety in dogs post-lockdown.

In response to pet owner feedback and the results of Survey 1, social media information interventions were provided via Facebook by CANZ (https://www.facebook.com/CompanionAnimalsNZ/, accessed on 10 March 2021) targeted at specific concerns that had been identified. Selected examples included the following:

(1) CANZ-Accredited Animal Behaviour Consultants Help with Lockdown Behaviour Problems—a 55-min-long livestream talking about behaviour problems related to lockdown. This reached 2494 news feeds with 610 people clicking on the post and 106 likes/comments/shares. The average video watch time was 0.39 s.
(2) Lockdown Behavioural Changes—a 7-min-long video talking about behaviour problems related to lockdown. Reached 3201 newsfeeds with 199 people clicking on the post and 97 likes/comments/shares. Average video watch time of 17 s.
(3) Figure 2 shows an example of a CANZ post. This example reached 11855 newsfeeds and had 33 shares, 138 likes, 19 emoji ‘loves’, 24 emoji ‘sads’, and 31 general comments.
(4) The “Eventually life will go back to normal” infographic (Figure 3) was CANZ’s most engaged with post with 110627 people being reached, 265 likes, 1765 loves, 282 comments, and 613 shares.

### 3.3. Survey 2—During Alert Level 1 (Post-Lockdown)

The survey was completed by 498 respondents. Table 1 summarises the percentage of pet types/s owned.

Table 6 shows the answer selected when respondents were asked: “Do you feel that your pets’ wellbeing was better during Alert Level 4 lockdown or during Alert Level 1?”. Forty-one percent (n = 204) of respondents felt their pets’ wellbeing was better during Alert Level 4 while 16.8% (n = 84) felt their pet’s wellbeing was better during Alert Level 1 post-lockdown.

Table 7 summarises the percentage of respondents that observed specified pet behaviours during Alert Level 4 (Lockdown) and Alert Level 1 (post-lockdown). Forty-three percent of respondents did not report any change in the pet behaviours listed at Alert Level 4, while 50% did not report any change in behaviour for Alert Level 1. The most frequently selected behaviour changes observed during Alert Level 4 were “Being calmer/more relaxed than normal” and “Being more affectionate than normal” with both these behaviours being observed by significantly more respondents when compared to Level 1 (Table 6). Conversely, significantly more respondents observed the following behaviours in Level 1 post-lockdown when compared to Level 4: “Being more needy/clingy than normal”, “Toileting issues” and “Showing signs of separation anxiety” (Table 6). Twelve percent (11.8%, n = 59) of respondents sought advice after Alert Level 4 for pet behavioural issues. Of these, 30.5% (n = 18) consulted with an animal behaviour professional, 22.0% (n = 13) sought advice from family and friends and 69.5% (n = 41) sought advice from other information sources such as the internet and social media.

When asked, “What, if anything, did you do to prepare your pet to transition from Alert Level 4 to Alert Level 1?”, 64.3% (n = 313) respondents wrote that they did nothing while 35.7% (n = 174) described how they prepared their pets. Similar percentages were observed if calculated for cat owning respondents and then dog owing respondents (30.8 and 39.8%, respectively). Many of those that prepared their pets for the transition gradually increased time away from their pets (n = 74), while some respondents either maintained their normal routine throughout the various alerts levels or gradually returned to their pre-lockdown routine before returning to work (n = 21), other respondents chose to work more hours from home (n = 21). Some of those returning to work noted deliberately spending more time with their pets when they were home (n = 12) and enriching their pets’ environment, for example, with toys and interactive treat feeders (n = 12). Dog owners also described gradual re-socialisation with other dogs and people (n = 20), gradual re-introduction to doggy daycare (n = 7) and hiring dog walkers (n =2).

Respondents were asked on a scale of 1 to 5 how much exercise have their pets been getting during Alert Level 1 post-lockdown compared with pre-lockdown (1 = A lot less, 5 = A lot more). The results are shown in Table 8, with 15.8% (n = 69) of respondents selecting 4 or 5 indicating their pets got more exercise during Alert Level 1 when compare with pre-lockdown, while 11.6% (n = 58) of selected 1 or 2 indicating they though their pets got less exercise. The majority of the respondents indicated that during Alert Level 1 (72.5%, n = 361) their pets exercise levels were similar to pre-lockdown. Similar results are obtained when the data from only those respondents owning dogs is analysed.

Using a Likert-like scale (1 = a lot less, 5 = a lot more), respondents were asked how much time they spent playing with their pets compared to pre-lockdown. The results are shown in Table 9, with 27.9% (n = 139) of respondents selecting 4 or 5 indicating they played more with their pets during Alert Level 1 post-lockdown compare to pre-lockdown, while 6.6% (n = 33) of selected 1 or 2 indicating they played with their pets less.

**Figure 4:**
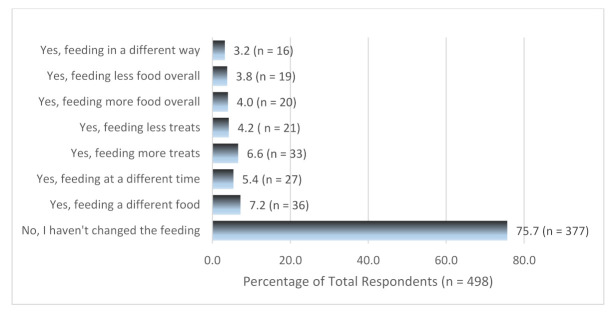
Feeding changes at Alert Level 1 post-lockdown compared with Alert Level 4.

Figure 4 shows how respondents changed how they feed their pets during Alert Level 1 post-lockdown compare to Alert Level 4. Most of the respondents (75.7%, n = 377) did not change the way they feed their pets post-lockdown. For those respondents that selected one of the options that indicated they did change the way they feed their pets, 116 explanations were provided. A content analysis revealed those feeding less overall food were doing so because their pets needed to lose weight gained over lockdown (n = 19). Health issues that arose during lockdown required diet changes for some pets (n = 11) as did life stage changes (puppy to adult, adult to senor diets; n = 5). Some respondents changed to cheaper pet food (n = 2), while others changed their pet’s diet due to availability (n = 10) or because they found a better diet option (n = 4). Some respondents reported changing feeding times to fit with their work schedules (n = 27), feeding more treats/food when at home either after work or due to working from home (n = 31), while other respondents noted they were feeding their pets less treats now they were back at work and not home as often (n = 10).

Of the 489 respondent that answered the question “Are you concerned about the impact of the COVID-19 pandemic on your pet’s wellbeing?”, 25.7% (n = 128) expressed that they were. Most of these respondents chose to explain what their concerns were. Content analysis identified that the most common concerns were that pets would miss having the company and attention they had during lockdown (n = 24) and experience separation anxiety (n = 15). Another common concern was the lack of access to animal or veterinary services and products during lockdown and the ongoing impact of this (n = 21). Specific to dogs were concerns about a lack of socialisation around other animals and people (n = 31) and the lack of off-lead exercise (n = 7). Other pet concerns mentioned included the impact of financial hardship (n = 2), the impact of routine changes (n = 8), pets being more needy/clingy (n = 5), pets being more anxious/jumpy around people (n = 4), toileting issues (n = 2), pets being overweight (n = 3), pets being COVID carriers (n = 2) and a lack of car sense (n = 1).

Sixty-seven (n = 326) percent of the 487 respondents also expressed that they were concerned about the impact of the COVID-19 pandemic on the wellbeing of New Zealand pets generally. An inductive thematic analysis of the descriptions of the concerns identified the following main themes: change of routine related issues (subthemes: missing company and affection, confusion, separation anxiety post-lockdown, pet acquire going into lockdown being neglected or abandoned/rehomed post-lockdown, less care of pets post lockdown including exercise, increased domestic violence/abuse during lockdown including pets), financial hardship related issues (subthemes: not being able to supply pet needs/neglect, increased human stress affecting pets, unable to afford veterinary and animal services and products, pet abandonment/rehoming), reduced access to veterinary and animal services and products (less desexing/more unwanted pregnancies, less grooming, feed changes), concern about pets carrying COVID-19, and the dog-specific issues due to lack of socialisation (eg due to lack of club activities, leashed walks only).

## 4. Discussion

The results from this study indicate that pets may have enjoyed improved welfare during the Alert Level 4 lockdown in New Zealand due to the possibility for increased human-pet interaction, with both adults and children spending a lot more time at home in line with the self-isolation directive from the Government. Most respondents could list at least one perceived benefit of lockdown for their pets. These included more company, more or better-quality attention (exercise, play, training, physical affection, grooming, treats, health surveillance/care, bonding), and environmental benefits (fewer cars on the road, less outside noise, more movement freedoms, and access to home comforts). Almost half of the respondents indicated that their pets got more exercise during lockdown and almost two-thirds of respondents indicated they spent more time playing with their pet. Just over half of the respondents thought that their pet’s wellbeing was better than usual during lockdown, with most of the remainder indicating that their pet’s wellbeing had not changed. Pet feeding practices were largely unchanged across the different alert levels; however, animals having gained weight was acknowledged as a common concern among respondents.

The findings of this study are consistent, in part, with a study of the effects of the Spanish COVID-19 lockdown on pets and their owners by Bowne and colleagues [8] which found the 57.3% of cat owners perceived their pets’ quality of life to be better during lockdown and 34.3% indicating that did not think their cat’s quality of life had changed. However, in contrast to our study, Bowne and colleagues [8] found that 62.1% of the dog owners surveyed thought their dog’s quality of life had got worse with a reduction in the number of walks and duration being reported. The Spanish lockdown was very similar in its restrictions (including those around dog walking) to the Alert Level 4 lockdown in New Zealand, but a notable difference between the two countries is that three-quarters of the Spanish respondents lived in apartments and the level of community transmission and thus the risk of infection was far greater in Spain. This may mean that while the restrictions on dog walking were similar in both countries, the level of impact of COVID-19 on dog walking may have been greater in Spain than in New Zealand. This is supported by Brown and colleagues reporting that before their lockdown, dogs went on an average of 3 walks per day, while during lockdown, they went on an average of 2.5 walks per day. By contrast, our study shows 49.7% of respondents with dogs were exercising them more than before lockdown. Our study also contrasted with the study by Christley and colleagues [16], which showed that in the UK during lockdown dogs were walked both less frequently and for shorter durations. However, this can likely be attributed to the difference in restrictions on outdoor exercise between New Zealand and the UK, where people were limited to one form of outdoor exercise per day.

Although the New Zealand Alert Level 4 lockdown was generally viewed as beneficial for pet wellbeing, just over half of the respondents identified negative impacts on their companion animals which included a reduction in the quality of dog exercise, disrupted routines, social and environmental changes, and health concerns. One driver of health concerns was less access to animal services and products. These results are consistent with the observations of Bowne and colleagues that found the most common concerns for dog owners was dog walking restrictions followed by loss of routine, and for cat owners, access to veterinary care and medication [8]. Approximately half of the New Zealand respondents noticed behavioural changes in their pets during lockdown, with pets commonly being described as more needy/clingy/attention-seeking, followed by pets being described as more affectionate. Similar numbers of respondents described pets as being calmer and more relaxed and/or happier compared to those that described their pets as making more noise, becoming more nervous/anxious/worried/stress and or being more demanding. The Spanish COVID-19 lockdown study by Bowne and colleagues also observed an increase in stress-associated behavioural changes, especially in the group of owners that were coping less well, implying that household stresses and people’s abilities to cope with them have an impact on pet wellbeing [8]. Morgan and colleagues also found there was an association between dog owners who felt their quality of life was impaired and the perceptions of reduced quality of life for their pet and the development of new behavioural problems [11]. Collectively, these findings are similar to those of a study on pet owners in the U.S. that focused on the negative impacts of COVID-19 on caring for pets, which concluded that pet owners experience unique COVID-related hardships, which need to be addressed in order to manage pet owner expectations, prevent problem pet behaviours, improve owner well-being and pet welfare [7].

Just over two-thirds of respondents expressed that they were concerned about their pet’s wellbeing after lockdown, with pets missing company/attention and separation anxiety being major themes. This level of concern is higher than that observed in the Spanish study, where approximately 40% of cat and dog owners surveyed were concerned their pet would not adapt to the situation after confinement ended [8]. In this study, respondents were also concerned about the wellbeing of New Zealand pets generally after lockdown. In addition to concerns about separation anxiety, loneliness and boredom, respondents were concerned about the impact of financial hardship on caring for animals and also an increase in unwanted and inadequately cared for animals.

Research has shown that companion animals can help some people cope with challenging situations and this was also found to be true during the COVID-19 lockdown [7,8,10,17]. However, the human-animal bond is complex and social exchange theory [18] has been used to explain this dynamic relationship, the product of which is the balance between perceived cost and benefits [8,10]. Globally many people chose to take on new pets going into lockdown [11,17,19]. Presumably, this decision was a consequence of the perceived social and health benefits outweighing the perceived costs of pet ownership. However, pets are a vulnerable sector of society as they are almost fully dependent on human care, and concerns about the long-term care of newly adopted animals have been raised in the literature [19]. Changing alert levels can alter the perceived costs and benefits of pet ownership [17], thus impacting on pet wellbeing. Vincent and colleagues highlight that the cost of caring for an animal during economic hardship or the burden of a daily care routine may result in the relinquishing of animals to a shelter both during and post-lockdown [17]. These pet-specific costs are potentially increased by animal behavioural issues that might have developed during lockdown or in groups of vulnerable people such as those infected with COVID-19, the elderly, those with mental health disorders or those with chronic illnesses, who might need additional support to care for their pets adequately. Thus, a One Welfare approach should be considered for the maintenance of the human-animal relationships formed or developed during the lockdown period. It has also been suggested in the academic literature that noticing and amplifying micro-practices that make a positive difference to non-human animals via social media may facilitate improve animal welfare practices [20].

In keeping with this, CANZ provided educational information via Facebook to address pet owner concerns, manage pet owner expectations and promote positive practices during the COVID-19 pandemic. Social networks allowed animal professionals to provide users with important COVID-19 related information impacting on animal welfare. To ensure user confidence, on 17 March 2020 the major social network companies declared that they would work closely together to combat false and misinformation and promote information from official sources [21]. Four levels of social network engagement have been acknowledged: (1) observer, (2) follower, (3) participant and (4) defender which can be further condensed into two levels of: passive engagement (level 2) and cognitive participative engagement (levels 3 and 4) [22]. Of the CANZ posts, infographics were engaged with by users more extensively than video resources. This contrasts with marketing research which reports that video posts perform best on Facebook [23]. COVID-19-specific research in Canada found that the type of embedded media used in Facebook posts was a determinant of engagement with public health communications, and that posts with simple, concise messages and high-quality media embedded, such as animations or infographics, were especially well shared [24]. Conversely, this research found that that extensive policy-related messages and video and infographics of lower quality were associated with less engagement.

Facebook users engaged with the CANZ infographics on the participatory level with likes, loves, shares, and comments. Compared to the infographics, the videos had less engagement and required longer viewing times (7–55 min) with the highest average watch time being 17 s. Interestingly, TikTok, a video sharing platform that has gained prominence during the COVID-19 pandemic uses a short-form format of 15 s [25]. Pérez-Escoda and colleagues highlight that social audiences need to be part of the media flow [22] which was reinforced by Raamkumar and colleagues, who observed that Facebook users engaging with content from health authorities were more likely to share a post aiding the dissemination of information than they were to react or comment [26]. It could be speculated that the anticipated uptake by one’s followers influences how users interact with content and in this respect those resources that require less time to view may be perceived as having better uptake by other social network users gaining more positive reactions (e.g., like, care, love), comments and shares. Further research into social network user engagement and uptake of information regarding companion animal wellbeing is needed.

In the Alert Level 1 survey, most respondents indicated that they thought their pet’s wellbeing was the same as it was in the Alert Level 4 lockdown or better. In Survey 1 more than half of the respondents thought their pet’s wellbeing was better during lockdown (Level 4) than it was pre-lockdown. It, therefore, suggests that Alert Level 1 life for many pets was perceived by their owners to be better than that of pre-lockdown. Half of the respondents did not observe any significant behaviour changes between Alert Level 4 and Alert Level 1 and just over one-third of respondents took steps to prepare their pets to transition out of lockdown with the internet and social media being a major source of information. It can, therefore, be speculated that pet-specific information provided via organisations such as CANZ using social media helped to re-adjust pet owner expectations and facilitate practices that resulted in pet-friendly Alert Level transitions.

In Survey 2, a quarter of the respondents expressed they were concerned about the impact of the COVID-19 pandemic on their pet’s wellbeing, significantly less than in Survey 1, suggesting that concerns had been mitigated. Interestingly, a similar percentage of respondents still expressed concern about the impact of the COVID-19 pandemic on the wellbeing of New Zealand pets generally, as did about the general impact of the lockdown on pets. Most concerns related to issues brought about by changes in routine (for both humans and their pets), financial hardship, and reduced access to veterinary and animal services and products. Dog-specific issues due to lack of socialisation were also highlighted as a concern. Again, this is reflective of the concerns of pet owners from other parts of the world [7,8]. There was very little concern about pets being involved in the transmission of COVID-19 among the New Zealand survey respondents which is in line with the current research evidence [27,28] and suggests accurate uptake of this information.

A significant determinant of an animal’s wellbeing is the balance between their experience of negative and positive affective states [29]. Several of the changes made by owners as they came out of lockdown, such as spending more time in play with their animal, working more from home, spending more time with pets, providing environmental enrichment, and employing dog walkers provide opportunities for pets to express positive emotions such as excitation and playfulness, the feeling of vitality and pleasure that comes with exercise, and the rewarding feelings that come from spending time with bonded humans. It is, therefore, reasonable to assume that at the time these changes were made the owners were enhancing their pet’s wellbeing. Further research is required to determine whether these changes and their associated positive effects on animal wellbeing, will be maintained long term.

### Limitations

Demographics were not collected therefore conclusions about sample representation cannot be made. Caution needs to be taken when extrapolating these results to all New Zealand pet owners as online surveys are typically taken by white women of higher socioeconomic status [30,31]. In these surveys, the respondents were free to answer questions taking all their pets into consideration as opposed to selecting only one of their pets as being the focus of their responses.

## 5. Conclusions

The results from this study indicate that pet owners perceived their pet’s wellbeing to be better during and after the Alert Level 4 lockdown in New Zealand when compared to pre-lockdown. This may have been due to improved welfare facilitated by the possibility for increased human-pet interaction along with the rapid increase in social media use providing animal professionals with the opportunity to promote positive human-pet interaction messages using a platform such as Facebook. The steps that were taken to prepare animals for a return to normal life may enhance pet wellbeing long-term if maintained.

## Figures and Tables

**Table 1:** The percentage of pet type/s owned in Survey 1 (n = 686) and survey 2 (n = 498).

Pet Type/s Owned	Survey 1		Survey 2	
	%	n	%	n
Dog/s total	84.0	576	74.7	372
Cat/s total	56.3	386	64.5	321
Dog/s and cat/s total	40.7	279	39.4	196
Dog/s only	36.7	252	29.7	148
Cat/s only	11.4	78	19.5	97
Cat/s and dog/s only	22.4	154	24.1	120
Dog/s and other (not cat)	6.6	45	5.6	28
Cat/s and other (not dog)	4.2	29	5.6	28
Cat/s, dog/s and other	22.4	154	15.3	76
Other (no dog/s or cat/s)	0.4	3	0.2	1

**Table 2:** Adult time at home compared to pre-lockdown determined using a Likert-like scale from 1 “No more than normal” through to 5 “Far more than before”.

Scale	Number of Respondents (n)	Percentage
1	37	5.4
2	22	3.2
3	61	8.9
4	118	17.2
5	448	65.3
Total	686	100

**Table 3:** Overall pet wellbeing during lockdown using a Likert-like scale from 1 “Wellbeing is far worse” through to 5 “Wellbeing is much better”.

Scale	Number of Respondents (n)	Percentage
1	9	1.3
2	42	6.1
3	260	37.9
4	229	33.4
5	146	21.3
Total	686	100

**Table 4:** How much exercise have their pets been getting during lockdown compared with pre-lockdown using a Likert-like scale from 1 “A lot less”, through to 5 “A lot more”.

Scale	Number of Respondents (n)	Percentage	Number of Dog Owning Respondents (n)	Percentage
1	15	2.2	12	2.1
2	71	10.4	63	10.9
3	293	42.6	215	37.3
4	193	28.1	180	31.3
5	114	16.6	106	18.4
Total	686	100	576	100

**Table 5:** How much have respondents played with their pets during lockdown compared with pre-lockdown using a Likert-like scale from 1 “A lot less”, through to 5 “A lot more”.

Scale	Number of Respondents (n)	Percentage
1	5	0.7
2	11	1.6
3	247	36.0
4	277	40.4
5	146	21.3
Total	686	100

**Table 6:** Alert Level 4 and 1 comparison of pet wellbeing.

Do You Feel That Your Pets’ Wellbeing was Better during Alert Level 4 Lockdown or during Alert Level 1?	Number of Respondents (n)	Percentage
Wellbeing is a bit better during Alert Level 1	36	7.2
Wellbeing is a lot better during Alert Level 1	48	9.6
Wellbeing is the same at Alert Level 4 and Alert Level 1	210	42.2
Wellbeing was a bit better during Alert Level 4	100	20.1
Wellbeing was much better during Alert Level 4	104	20.9
Total	498	100

**Table 7:** Percentage of respondents that observed specified pet behaviours during Alert Level 4 (Lockdown) and Alert Level 1 (post-lockdown).

Behaviour	Alert Level 4 (Lockdown)		Alert Level 1 (Post-Lockdown)	
	No. Respondents That Observed the Behaviour	% Total Respondents (n = 498)	No. Respondents That Observed the Behaviour	% Total Respondents (n = 498)
Being more needy/clingy than normal	67	13.5 *^a^*	114	22.9 *^b^*
Being more affectionate than normal	113	22.7 *^a^*	71	14.3 *^b^*
Being calmer/more relaxed than normal	145	29.1 *^a^*	34	6.8 *^b^*
Making more noise than normal	46	9.2 *^a^*	60	12.0 *^a^*
Being more nervous/anxious/worried/ stressed than normal	24	4.8 *^a^*	39	7.8 *^a^*
Being sad/depressed/lethargic	11	2.2 *^a^*	18	3.6 *^a^*
Being bored	49	9.8 *^a^*	44	8.8 *^a^*
Being more reactive to/less sociable with other animals	28	5.6 *^a^*	36	7.2 *^a^*
Toileting issues	7	1.4 *^a^*	18	3.6 *^b^*
Showing signs of separation anxiety	20	4.0 *^a^*	65	13.1 *^b^*
None of these behaviours	215	43.2 *^a^*	249	50.0 *^b^*

^*a*,*b*^ Different superscript letters denote column percentages for Levels 4 and 1 are significantly from each other at the 0.05level. No. = Number.

**Table 8:** How much exercise have pets been getting during Alert Level 1 (post-lockdown) compared with pre-lockdown using a Likert-like scale from 1 “A lot less”, through to 5 “A lot more”.

Scale	Number of Respondents (n)	Percentage	Number of Dog Owning Respondents (n)	Percentage
1	10	2.0	8	2.2
2	48	9.6	45	12.1
3	361	72.5	247	66.4
4	58	11.6	53	14.2
5	21	4.2	19	5.1
Total	498	100	372	100

**Table 9:** How much have respondents played with their pets during Alert Level 1 (post-lockdown) compared with pre-lockdown using a Likert-like scale from 1 “A lot less”, through to 5 “A lot more”.

Scale	Number of Respondents (n)	Percentage
1	3	0.6
2	30	6.0
3	326	65.5
4	111	22.3
5	28	5.6
Total	498	100

## Data Availability

The data presented in this study are available on request from the corresponding author.

## Supplementary Materials

The following are available online at https://www.mdpi.com/2076-2615/11/3/758/s1, Figure S1: Survey 1 Pet Wellbeing During COVID-19 Lockdown, Figure S2: Survey 2 The Impact of COVID-19 pandemic on the Wellbeing of NZ Pets.
