# Supplementary material for: Locking down the Impact of New Zealand’s COVID-19 Alert Level Changes on Pets
Source: Animals (Basel). 2021 Mar 10;11(3):758. doi: 10.3390/ani11030758 (PMC8000179; doi:10.3390/ani11030758)
Supplement: Supplementary file 1 [file animals-11-00758-s001.pdf]

Supplementary Material

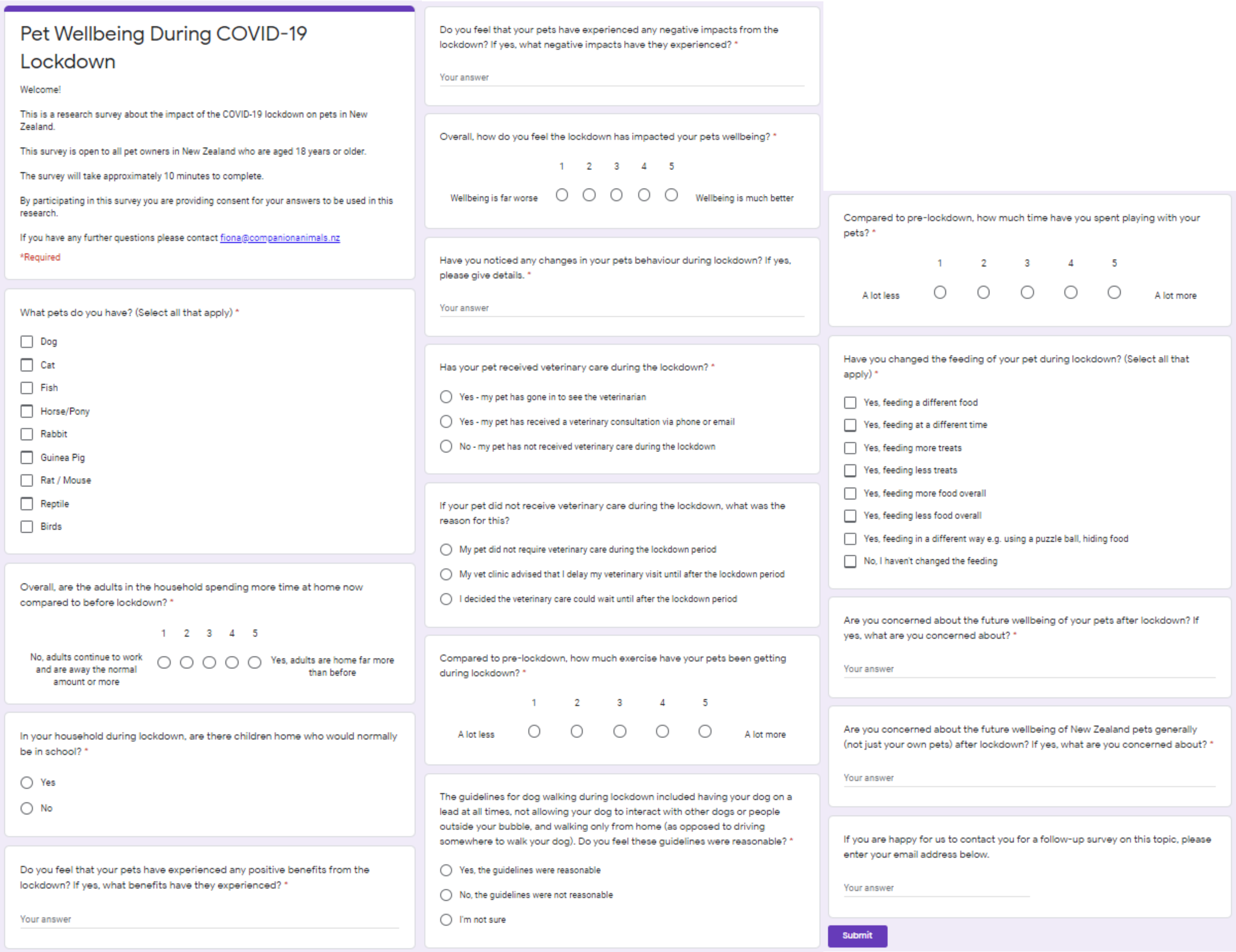

Pet Wellbeing During COVID-19 Lockdown

Welcome!

This is a research survey about the impact of the COVID-19 lockdown on pets in New Zealand.

This survey is open to all pet owners in New Zealand who are aged 18 years or older.

The survey will take approximately 10 minutes to complete.

By participating in this survey you are providing consent for your answers to be used in this research.

If you have any further questions please contact fiona@companionanimals.nz

*Required

What pets do you have? (Select all that apply) *

- Dog
- Cat
- Fish
- Horse/Pony
- Rabbit
- Guinea Pig
- Rat / Mouse
- Reptile
- Birds

Overall, are the adults in the household spending more time at home now compared to before lockdown? *

No, adults continue to work and are away the normal amount or more 1 2 3 4 5 Yes, adults are home far more than before

In your household during lockdown, are there children home who would normally be in school? *

- Yes
- No

Do you feel that your pets have experienced any positive benefits from the lockdown? If yes, what benefits have they experienced? *

Your answer

Do you feel that your pets have experienced any negative impacts from the lockdown? If yes, what negative impacts have they experienced? *

Your answer

Overall, how do you feel the lockdown has impacted your pets wellbeing? *

Wellbeing is far worse 1 2 3 4 5 Wellbeing is much better

Have you noticed any changes in your pets behaviour during lockdown? If yes, please give details. *

Your answer

Has your pet received veterinary care during the lockdown? *

- Yes - my pet has gone in to see the veterinarian
- Yes - my pet has received a veterinary consultation via phone or email
- No - my pet has not received veterinary care during the lockdown

If your pet did not receive veterinary care during the lockdown, what was the reason for this?

- My pet did not require veterinary care during the lockdown period
- My vet clinic advised that I delay my veterinary visit until after the lockdown period
- I decided the veterinary care could wait until after the lockdown period

Compared to pre-lockdown, how much exercise have your pets been getting during lockdown? *

A lot less 1 2 3 4 5 A lot more

The guidelines for dog walking during lockdown included having your dog on a lead at all times, not allowing your dog to interact with other dogs or people outside your bubble, and walking only from home (as opposed to driving somewhere to walk your dog). Do you feel these guidelines were reasonable? *

- Yes, the guidelines were reasonable
- No, the guidelines were not reasonable
- I'm not sure

Compared to pre-lockdown, how much time have you spent playing with your pets? *

A lot less 1 2 3 4 5 A lot more

Have you changed the feeding of your pet during lockdown? (Select all that apply) *

- Yes, feeding a different food
- Yes, feeding at a different time
- Yes, feeding more treats
- Yes, feeding less treats
- Yes, feeding more food overall
- Yes, feeding less food overall
- Yes, feeding in a different way e.g. using a puzzle ball, hiding food
- No, I haven't changed the feeding

Are you concerned about the future wellbeing of your pets after lockdown? If yes, what are you concerned about? *

Your answer

Are you concerned about the future wellbeing of New Zealand pets generally (not just your own pets) after lockdown? If yes, what are you concerned about? *

Your answer

If you are happy for us to contact you for a follow-up survey on this topic, please enter your email address below.

Your answer

Submit

Figure S1. Survey 1 Pet Wellbeing During COVID-19 Lockdown.

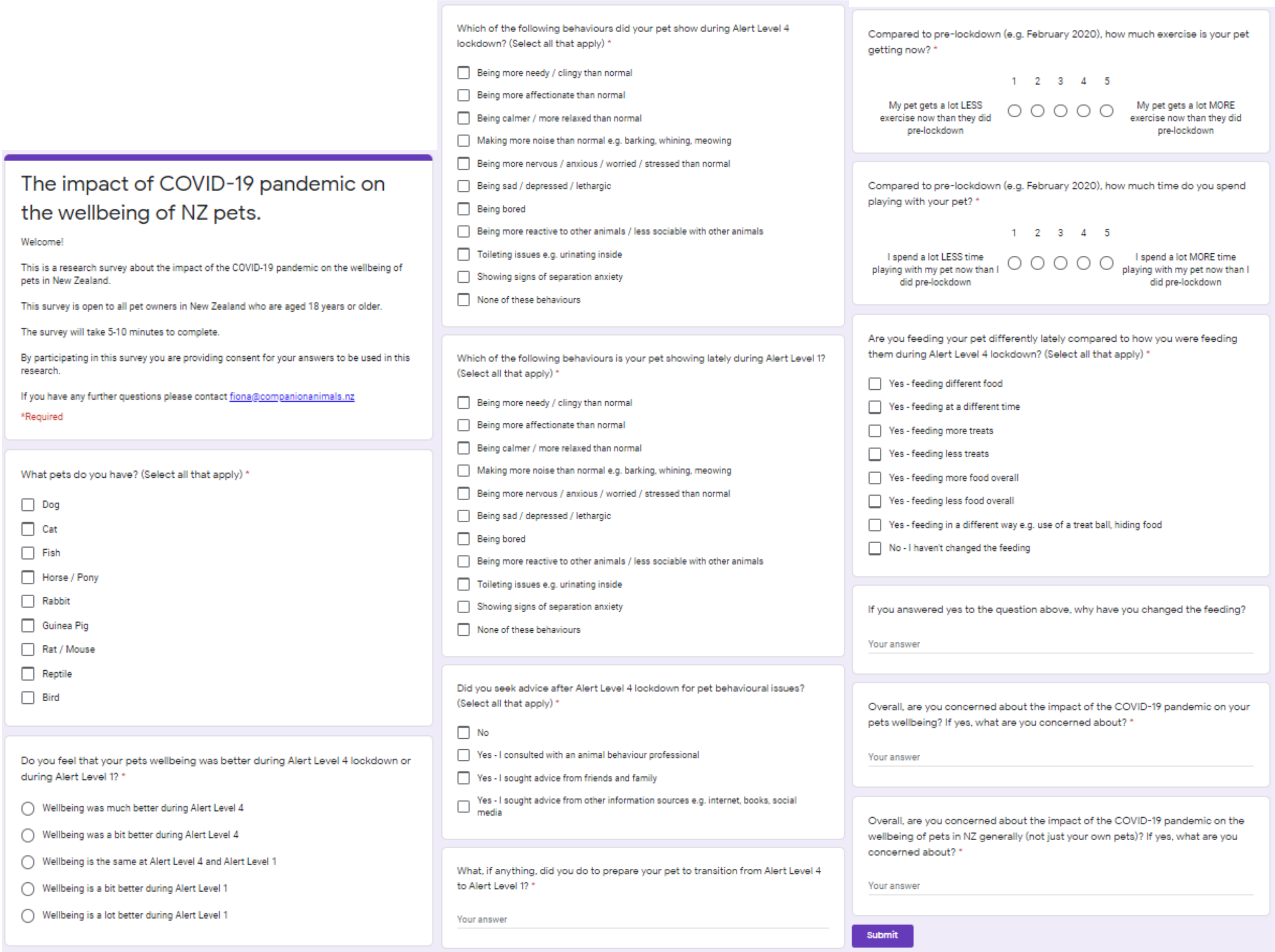

The impact of COVID-19 pandemic on the wellbeing of NZ pets.

Welcome!

This is a research survey about the impact of the COVID-19 pandemic on the wellbeing of pets in New Zealand.

This survey is open to all pet owners in New Zealand who are aged 18 years or older.

The survey will take 5-10 minutes to complete.

By participating in this survey you are providing consent for your answers to be used in this research.

If you have any further questions please contact fiona@companionanimals.nz

*Required

What pets do you have? (Select all that apply) *

- [ ] Dog
- [ ] Cat
- [ ] Fish
- [ ] Horse / Pony
- [ ] Rabbit
- [ ] Guinea Pig
- [ ] Rat / Mouse
- [ ] Reptile
- [ ] Bird

Do you feel that your pets wellbeing was better during Alert Level 4 lockdown or during Alert Level 1? *

- ○ Wellbeing was much better during Alert Level 4
- ○ Wellbeing was a bit better during Alert Level 4
- ○ Wellbeing is the same at Alert Level 4 and Alert Level 1
- ○ Wellbeing is a bit better during Alert Level 1
- ○ Wellbeing is a lot better during Alert Level 1

Which of the following behaviours did your pet show during Alert Level 4 lockdown? (Select all that apply) *

- [ ] Being more needy / clingy than normal
- [ ] Being more affectionate than normal
- [ ] Being calmer / more relaxed than normal
- [ ] Making more noise than normal e.g. barking, whining, meowing
- [ ] Being more nervous / anxious / worried / stressed than normal
- [ ] Being sad / depressed / lethargic
- [ ] Being bored
- [ ] Being more reactive to other animals / less sociable with other animals
- [ ] Toileting issues e.g. urinating inside
- [ ] Showing signs of separation anxiety
- [ ] None of these behaviours

Which of the following behaviours is your pet showing lately during Alert Level 1? (Select all that apply) *

- [ ] Being more needy / clingy than normal
- [ ] Being more affectionate than normal
- [ ] Being calmer / more relaxed than normal
- [ ] Making more noise than normal e.g. barking, whining, meowing
- [ ] Being more nervous / anxious / worried / stressed than normal
- [ ] Being sad / depressed / lethargic
- [ ] Being bored
- [ ] Being more reactive to other animals / less sociable with other animals
- [ ] Toileting issues e.g. urinating inside
- [ ] Showing signs of separation anxiety
- [ ] None of these behaviours

Did you seek advice after Alert Level 4 lockdown for pet behavioural issues? (Select all that apply) *

- [ ] No
- [ ] Yes - I consulted with an animal behaviour professional
- [ ] Yes - I sought advice from friends and family
- [ ] Yes - I sought advice from other information sources e.g. internet, books, social media

What, if anything, did you do to prepare your pet to transition from Alert Level 4 to Alert Level 1? *

Your answer

Compared to pre-lockdown (e.g. February 2020), how much exercise is your pet getting now? *

	1	2	3	4	5	
My pet gets a lot LESS exercise now than they did pre-lockdown	○	○	○	○	○	My pet gets a lot MORE exercise now than they did pre-lockdown

Compared to pre-lockdown (e.g. February 2020), how much time do you spend playing with your pet? *

	1	2	3	4	5	
I spend a lot LESS time playing with my pet now than I did pre-lockdown	○	○	○	○	○	I spend a lot MORE time playing with my pet now than I did pre-lockdown

Are you feeding your pet differently lately compared to how you were feeding them during Alert Level 4 lockdown? (Select all that apply) *

- [ ] Yes - feeding different food
- [ ] Yes - feeding at a different time
- [ ] Yes - feeding more treats
- [ ] Yes - feeding less treats
- [ ] Yes - feeding more food overall
- [ ] Yes - feeding less food overall
- [ ] Yes - feeding in a different way e.g. use of a treat ball, hiding food
- [ ] No - I haven't changed the feeding

If you answered yes to the question above, why have you changed the feeding?

Your answer

Overall, are you concerned about the impact of the COVID-19 pandemic on your pets wellbeing? If yes, what are you concerned about? *

Your answer

Overall, are you concerned about the impact of the COVID-19 pandemic on the wellbeing of pets in NZ generally (not just your own pets)? If yes, what are you concerned about? *

Your answer

Submit

Figure S2. Survey 2 The Impact of COVID-19 pandemic on the Wellbeing of NZ Pets.
